# Radiation exposure for pedicle screw placement with three different navigation system and imaging combinations in a sawbone model

**DOI:** 10.1186/s12891-023-06880-2

**Published:** 2023-09-23

**Authors:** Nils Beisemann, Jula Gierse, Eric Mandelka, Frank Hassel, Paul A. Grützner, Jochen Franke, Sven Y. Vetter

**Affiliations:** 1 Research Group Medical Imaging and Navigation in Trauma and Orthopedic Surgery (MINTOS), BG Klinik (BG Trauma Center) Ludwigshafen, Ludwig-Guttmann-Strasse 13, 67071 Ludwigshafen, Germany; 2 Department of Spine Surgery, Loretto Hospital, Mercystrasse 6, 79100 Freiburg Im Breisgau, Germany

**Keywords:** Spinal navigation, 3D imaging, Radiation exposure, Dorsal instrumentation

## Abstract

**Background:**

Studies have shown that pedicle screw placement using navigation can potentially reduce radiation exposure of surgical personnel compared to conventional methods. Spinal navigation is based on an interaction of a navigation software and 3D imaging. The 3D image data can be acquired using different imaging modalities such as iCT and CBCT. These imaging modalities vary regarding acquisition technique and field of view. The current literature varies greatly in study design, in form of dose registration, as well as navigation systems and imaging modalities analyzed.

Therefore, the aim of this study was a standardized comparison of three navigation and imaging system combinations in an experimental setting in an artificial spine model.

**Methods:**

In this experimental study dorsal instrumentation of the thoracolumbar spine was performed using three imaging/navigation system combinations. The system combinations applied were the iCT/Curve, cCBCT/Pulse and oCBCT/StealthStation. Referencing scans were obtained with each imaging modality and served as basis for the respective navigation system. In each group 10 artificial spine models received bilateral dorsal instrumentation from T11-S1. 2 referencing and control scans were acquired with the CBCTs, since their field of view could only depict up to five vertebrae in one scan. The field of view of the iCT enabled the depiction of T11-S1 in one scan. After instrumentation the region of interest was scanned again for evaluation of the screw position, therefore only one referencing and one control scan were obtained. Two dose meters were installed in a spine bed ventral of L1 and S1. The dose measurements in each location and in total were analyzed for each system combination. Time demand regarding screw placement was also assessed for all system combinations.

**Results:**

The mean radiation dose in the iCT group measured 1,6 ± 1,1 mGy. In the cCBCT group the mean was 3,6 ± 0,3 mGy and in the oCBCT group 10,3 ± 5,7 mGy were measured. The analysis of variance (ANOVA) showed a significant (*p* < 0.0001) difference between the three groups. The multiple comparisions by the Kruskall-Wallis test showed no significant difference for the comparison of iCT and cCBCT (*p*^1^ = 0,13). Significant differences were found for the direct comparison of iCT and oCBCT (*p*^2^ < 0,0001), as well as cCBCT and oCBCT (*p*^3^ = 0,02). Statistical analysis showed that significantly (iCT vs. oCBCT *p* = 0,0434; cCBCT vs. oCBCT *p* = 0,0083) less time was needed for oCBCT based navigated pedicle screw placement compared to the other system combinations (iCT vs. cCBCT *p* = 0,871).

**Conclusion:**

Under standardized conditions oCBCT navigation demanded twice as much radiation as the cCBCT for the same number of scans, while the radiation exposure measured for the iCT and cCBCT for one scan was comparable. Yet, time effort was significantly less for oCBCT based navigation. However, for transferability into clinical practice additional studies should follow evaluating parameters regarding feasibility and clinical outcome under standardized conditions.

## Background

Minimally invasive treatment techniques for dorsal stabilization of the spine are frequently preferred since they show advantages in certain indications over open procedures. The application of intraoperative navigation facilitates the implementation of these techniques. Advantages of intraoperative 3D imaging in combination with navigation systems in terms of better visualization of complex anatomic structures are evident [1, 2]. 3D navigation can be based on different intraoperative imaging modalities, that differ in various aspects such as image quality and field of view. Modalities available include cone beam computed tomography (CBCT) or intraoperative computed tomography (iCT) [3–6]. Advantages of the iCT compared to the CBCT are higher image resolution and a lager field of view [7]. The design of the CBCT permits a higher mobility, and thus allows a greater flexibility in use. An additional device for standard 2D image acquisition is needed if navigation is based on iCT, whereas the CBCT is able to obtain both formats (2D and 3D images), which facilitates a more precise targeting of the region of interest and thus reduces radiation exposure [8–10]. Within the group of available CBCT systems there are differences in gantry size and form (O-arm vs. C-arm) as well as detector technology (image-intensifier vs. flat panel technology) [7]. These imaging modalities are paired with a compatible navigation system software. Although theoretically any imaging modality could be matched with any navigation system, combinations by the same manufacturer are frequently used [11].

Studies show that by navigated pedicle screw placement the radiation exposure of the OR staff can potentially be reduced compared to conventional fluoroscopy guided techniques [12, 13]. The comparative evaluation of different studies on radiation exposure proves difficult since study settings, registration of dose and systems analyzed vary greatly [14–17].

Therefore, the aim of this study was the comparison of the radiation exposure of three imaging systems in a standardized experimental setting in an artificial bone model for navigated dorsal instrumentation of the thoracolumbar spine.

## Methods

### Study design

**Fig. 1:**
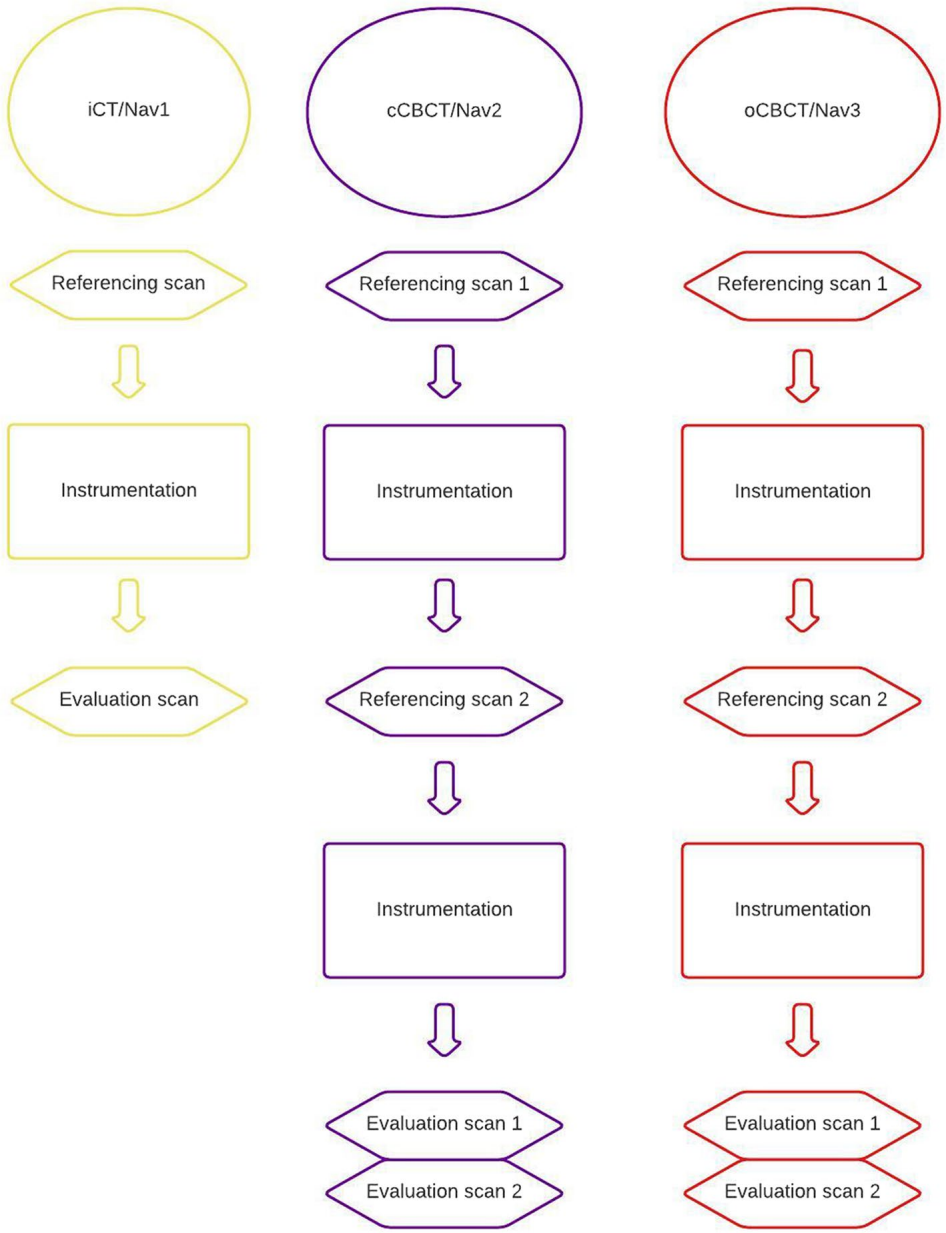
Study design. In the cCBCT and oCBCT group two scans had to be performed for referencing as well as evaluation due to the smaller field of view

**Fig. 2:**
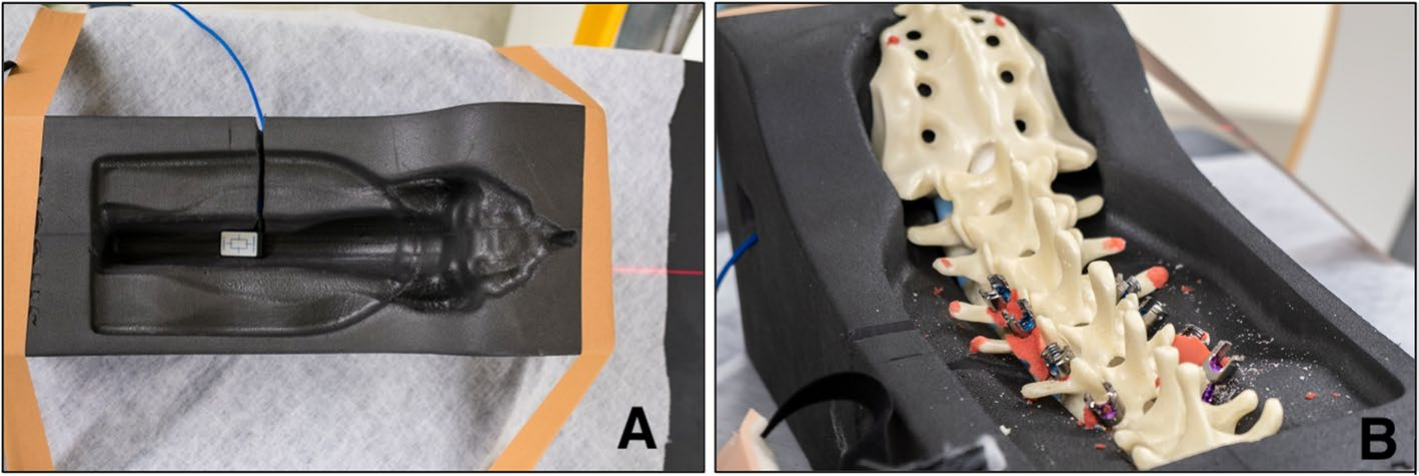
**A** Spine bed with sensor integrated at L1 (**B**) artificial spine model with removed muscle imitation in spine bed

This experimental study was composed of three groups, based on the combination of imaging modality and navigation system (Fig. 1). In the iCT group the Airo (Brainlab AG, Munich, Germany) and Curve navigation (Brainlab AG, Munich, Germany) were used. In the cCBCT group the Cios Spin C-arm (Siemens, Forchheim, Germany) was combined with the Pulse navigation system (NuVasive, San Diego, California, USA). For the oCBCT group, the O-arm (Medtronic, Minneapolis, USA) was used in combination with the Stealth Station (Medtronic, Minneapolis, USA) navigation system. In every group 10 identical radiopaque artificial spine models (spine model nr. LSS9370.0; model skin nr. PR1549.30; spine bed nr. PR1309; Synbone AG, Zizers, Switzerland) received dorsal instrumentation from T11 to S1, which resulted in 160 screws planned per group and a total of 480 planned screws (Reline MAS, NuVasive, San Diego, California, USA). The complete model consisting of spine bed, artificial spine with muscles and synthetic skin was fixed to an operating table in prone position (Fig. 2). 3D imaging scans were obtained before instrumentation as referencing scans as basis for navigation and after instrumentation for evaluation of the final screw position. T11-S1 could not be captured in one scan with the CBCT since the field of view is smaller than that of the iCT. Subsequently, two referencing and evaluation scans had to be acquired in the C-arm CBCT (cCBCT) and O-arm CBCT (oCBCT) group, whereas only one referencing and evaluation scan had to be acquired in the iCT group for the purpose of the instrumentation of the eight vertebrae.

For all devices, 3D imaging was performed with the standard radiation protocols (iCT: adult lumbar spine, patient weight 70 kg; slice thickness 1mm; C-arm CBCT: standard quality, 200 images in 30 s; image resolution (mm): 0,3125 × 0,3125 × 0,3125; slice thickness: 0,313mm; CBCT: patient size medium; image resolution (mm): 0,415 × 0,415 × 0,833; slice thickness: 0,833mm).

### Radiation registration

The Conny II constancy dosemeter (PTW, Freiburg, Germany) was used for a standardized comparison of the radiation dose for the different system combinations. The Conny II measures absorbed dose and dose rate at 30kV and 70/100kV and displays the units air kerma (Gy), air kerma rate (Gy/s) and radiation time (s) on an integrated display. The same two Conny II dosemeters were used in each group of the study. The sensors for measuring radiation dose (in mGy) were installed in fixed locations in the spine bed in which the spine models were placed. One sensor was placed ventral to the spine at the level of L1 (Fig. 2A) and the second sensor was placed caudal in the area of the bladder or female reproductive organs according to the localizations most commonly used in literature [18, 19].

**Fig. 3:**
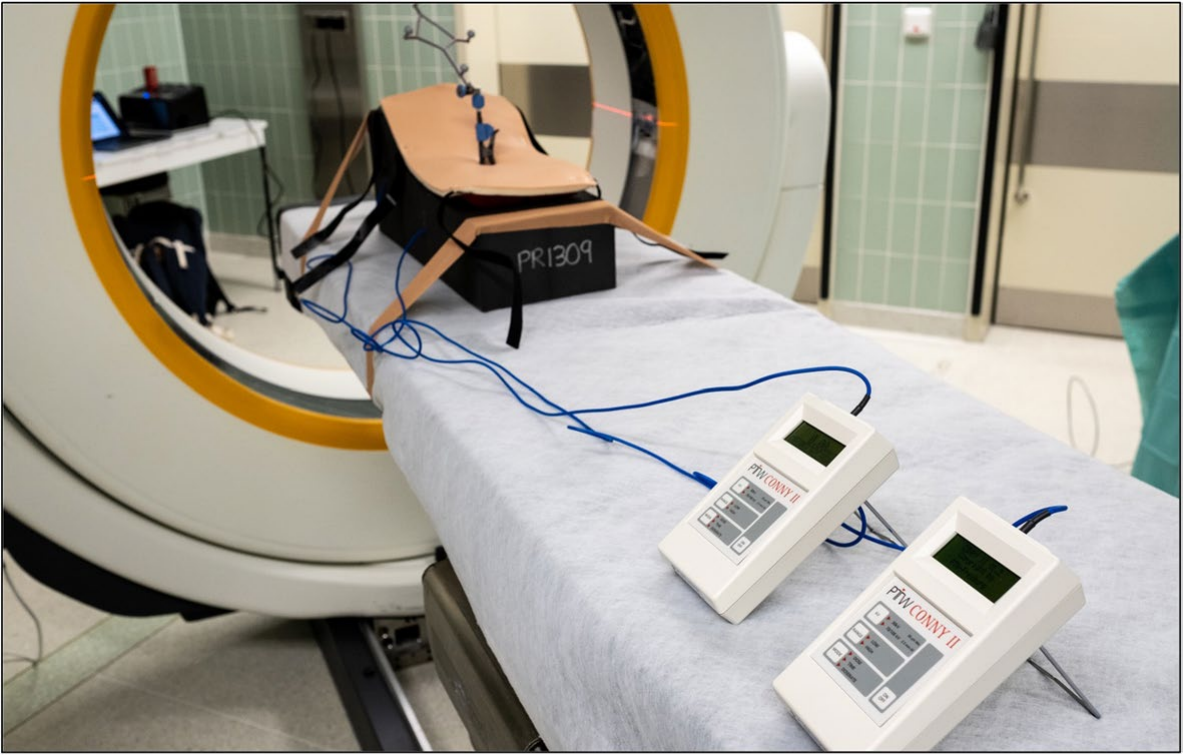
Experimental setup of model in prone position and Conny II devices

Figure 2B shows the instrumented screws in the artificial spine after removal of the synthetic muscles and skin. The dosemeter displays were placed cranial to the model (Fig. 3).

The Conny II complies with the standards of the IEC (International Electrotechnical Committee) standard, IEC 61674. All individual values measured in one location within one experiment were added for statistic evaluation.

In addition, time effort for the placement of the respective screws was assessed and compared between the system combinations. Time effort was determined with a stopwatch. The first incision in the artificial skin was selected as the starting point of the surgical time, the end point was determined as the moment after the final scans had been completed.

### Statistics

All data were tested for gaussian distribution using the Kolmogorow-Smirnow test. Analysis of radiation dose was performed by applying an ANOVA to test for difference between all three groups (Kruskall-Wallis test). Means and standard deviation (SD) were determined and multiple comparisions were conducted with the Kruskall-Wallis test for all three group combinations. Time effort was analyzed using the one-way ANOVA and each pair was compared using Welch’s t test. The significance level was set at *p* < 0.05. Statistical analysis of data tabulated in Excel (Microsoft Excel 2019, version 16.38) was performed using JMP version 14.2.0 (SAS, Cary, USA). Figures were created using Prism 8 (Graphpad Software, San Diego, USA).

## Results

All three system combinations were compared by the ANOVA, which showed a significant (*p* < 0.0001) difference in terms of mean radiation dose measured.

Table 1 shows the mean radiation dose and SD measured, as well as the statistical comparison between the group combinations by Kruskall-Wallis test.

**Table 1:** Mean and SD of radiation dose per group and comparison of mean values *p*^1^: iCT vs. cCBCT, *p*2: iCT vs. oCBCT, *p*^3^: cCBCT vs. oCBCT *sig

	iCT	cCBCT	oCBCT	
Radiation dose [mGy]	1,6 ± 1,1	3,6 ± 0,3	10,3 ± 5,7	*p*^1^ = 0,13 *p*^2^ < 0,0001* *p*^3^ = 0,02*

**Fig. 4:**
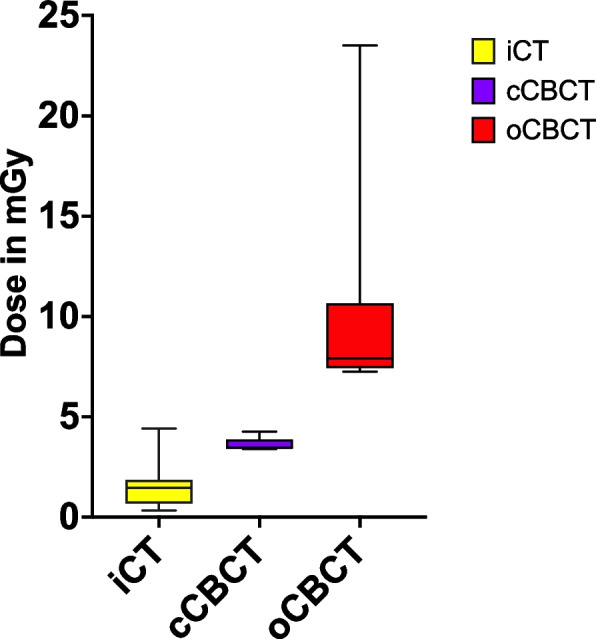
Average radiation dose measured per group

Figure 4 shows the average measured radiation per instrumentation for the different groups.

**Fig. 5:**
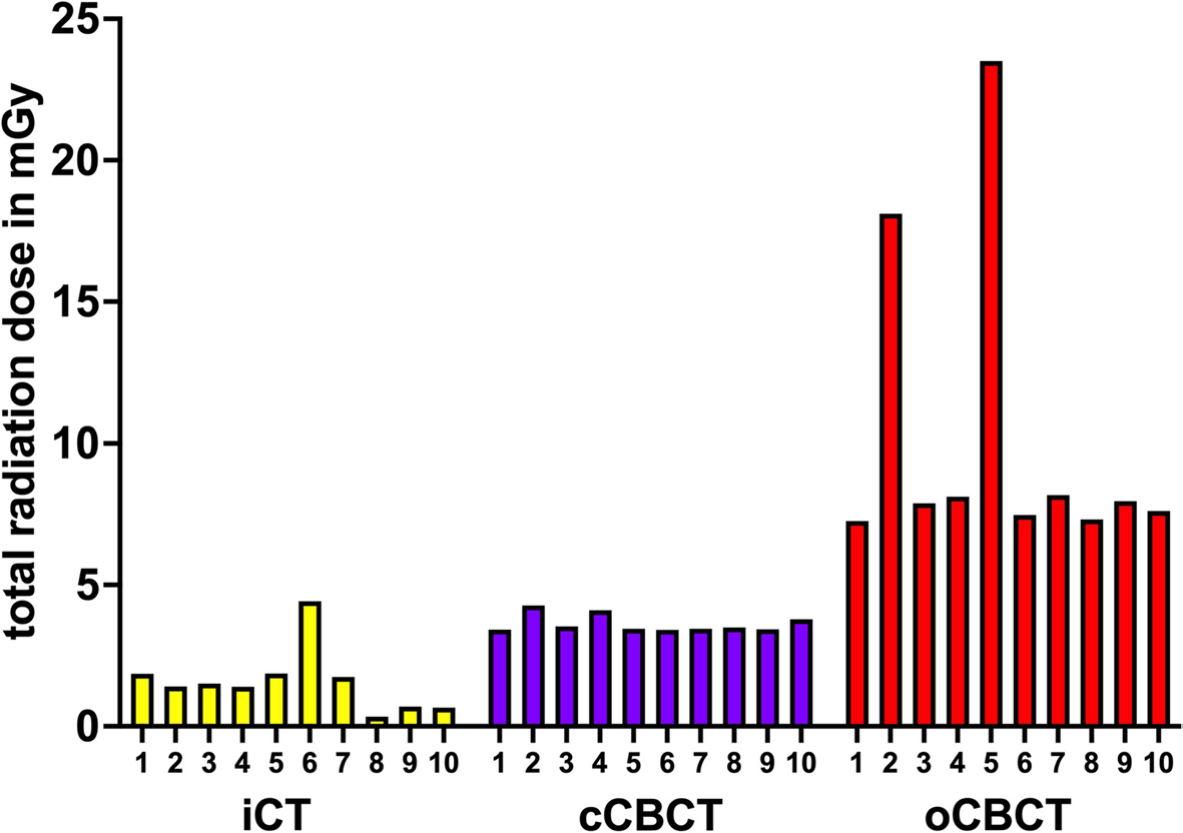
Total radiation dose recorded per instrumentation in each group

Figure 5 depicts the overall measured radiation dose per spine instrumentation in each group.

**Fig. 6:**
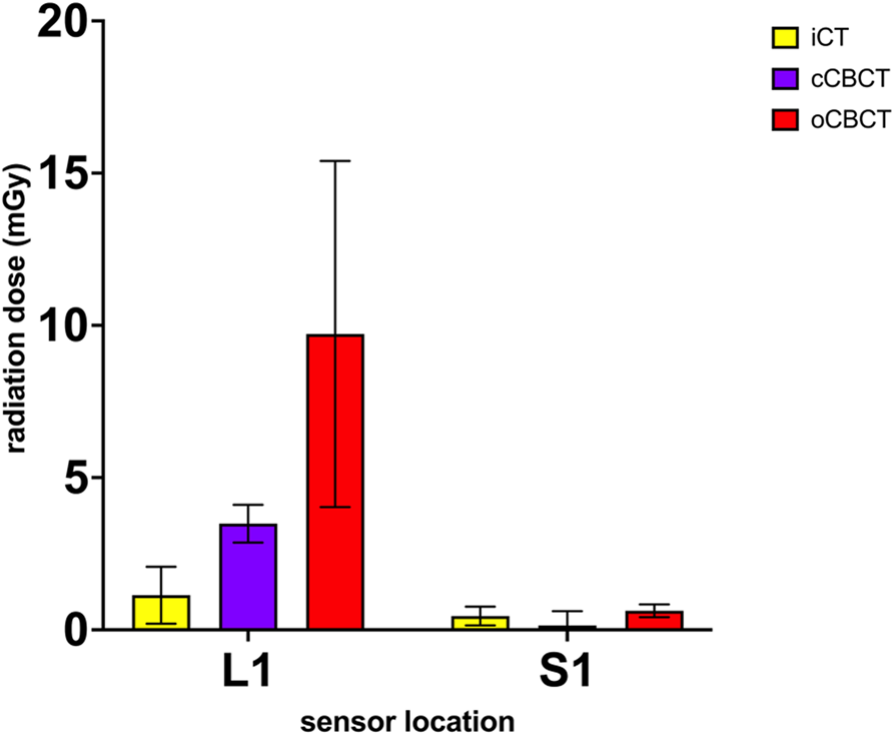
Average radiation dose measured per location in each group

Figure 6 shows the mean and SD radiation dose measured by each installed sensor per group. Mean dose and SD are listed in Table 2.

**Table 2:** Mean and SD per sensor location for each system

	**iCT**		**cCBCT**		**oCBCT**	
**Sensor location**	L1	S1	L1	S1	L1	S1
**Mean in mGy**	1,137	0,454	3,487	0,148	9,717	0,625
**SD in mGy**	0,9340	0,308	0,6214	0,467	5,685	0,210

The one-way ANOVA comparing time effort between the three system combinations concerning dorsal instrumentation showed a significant difference (*p* = 0,02).

Table 3 shows mean and SD, as well as the statistical comparison between the group combinations by Welch’s t test.

**Table 3:** Mean and SD of time effort per group and comparison of mean values *p*^1^: iCT vs. cCBCT, *p*2: iCT vs. oCBCT, *p*^3^: cCBCT vs. oCBCT *sig

	iCT	cCBCT	oCBCT	
Time effort in min	51,3 ± 12	52 ± 6,9	40,6 ± 9,9	*p*^1^ = 0,8710 *p*^2^ = 0,0434* *p*^3^ = 0,0083*

## Discussion

This study aimed to investigate and compare the radiation exposure of three imaging and navigation system combinations during dorsal instrumentation in a standardized experimental setting. The results showed a significantly higher radiation dose measured if images were obtained by the oCBCT, while radiation exposure of iCT and cCBCT is comparable. The main fraction of radiation was measured at the L1 dosemeter in all groups. Time effort was significantly less for the oCBCT trials compared to the iCT and cCBCT based system combinations.

Regarding methodology the authors designed the study to allow a direct comparison of the system combinations. The installation of the sensors in the spine bed, ensured that the radiation dose was always measured in the exact same location, in the exact same fashion and measuring unit. The internal radiation protocols of the imaging devices record the radiation emitted for image data acquisition. Depending on the imaging modality different units are used in these protocols, therefore, the values of these protocols are not directly comparable. Moreover, these protocols do not reflect how much radiation the patient is exposed to. The patient-specific radiation exposure possibly differs for the different imaging modalities. Also, in this study radiation emitted by 2D fluoroscopy and 3D scans had to be registered and considered. Therefore, dosemeters (Conny II, PTW, Freiburg, Germany) were utilized to register the total dose in identical units (mGy). The determination of the dose in mGy was deemed most sensible and sufficient for the direct comparison of the device combinations in this setting. Tonetti et al. also determined the dose in Gy to compare devices, since deterministic radiation consequences are dependent on the absorbed radiation dose in Gy [13]. Other studies assessed the organ dose in Sv [8, 20–22]. Dose and organ dose can be converted into one another using coefficients depending on the tissue and radiation type, thus results may contingently be compared. The authors also decided against organ dose as primary parameter, since artificial bone models, subsequently missing human tissue, were the subject of this study. The locations of the sensors were chosen based on results of studies on radiation dose during intraoperative 2D and 3D imaging of spinal procedures, which assessed the highest organ dose in the small intestine and red bone marrow region in the iliosacral joint and gonads [14, 18, 23, 24].

The results showed that the use of oCBCT based navigation entailed twice the radiation exposure compared to cCBCT based navigation although the same number of scans was performed. A possible explanation may be a missing regulatory mechanism of the oCBCT, that reduces dose if it is not required. Nonetheless, the use of the oCBCT based navigation is considered overall safe according to the Radiation Protection Guidelines. The registered radiation dose differed among the installed sensors. The main fraction of radiation was measured at the L1 level in all groups, yet this effect was more explicit in the cCBCT and oCBCT group. In these groups two scans had to be obtained to display the complete spine segment for instrumentation. L1 was included in the cranial scan and at the edge of the caudal scan or respectively the level adjacent to the caudal scan, which explains the higher dose values for this location. The lowest radiation exposure was measured in for the application of the iCT, which might be affected by the sensor location, as well as technical aspects of the image acquisition. The total radiation dose measured per instrumentation was rather consistent in all groups. The highest standard deviation presented in the oCBCT group, which can be explained by the intensity of radiation and the angle with which the rays hit the sensors. This is influenced by the positioning of the imaging unit in relation to the patient or as in this case, the sensors. The variance in value shows the sensitivity with which this data was collected.

In their review Pennington et al. analyzed radiation exposure of patient and surgical staff. They concluded that image-guided procedures apply less radiation to the patient than a CT of the lumbar spine. They suggest that the use of iCT and oCBCT-navigation reduces overall radiation exposure of the patient compared with conventional methods [25]. In addition, they justified any occurring radiation exposure by the increased accuracy of screw placement [25]. Tonetti et al. demonstrated that radiation exposure to the patient may be reduced when navigation is applied, since according to their study a postoperative CT is not necessary in every case [12, 13]. Although it should be considered that radiation exposure and application with any device varies in correlation with the dose protocol chosen based on the patient's constitution, furthering the need for a standardized study setting [26].

Foster et al. compared the same imaging modalities (iCT (Airo) vs. cCBCT (Cios Spin) vs. oCBCT (O-arm)) regarding radiation exposure that were used in the present study. In contrast, their study design only entailed the single instrumentation of L4 and L5 in one fresh torso cadaver without the use of navigation. The placed screws were then depicted multiple times with the different devices under the application of different dose protocols. In their analysis they evaluated a combination of image quality and radiation exposure. Their results of the effective dose for the application of the medium protocol with all three devices concur with the results of the present study [27].

Scarone et al. used the same iCT and oCBCT systems as in this study and measured significantly lower radiation exposure by iCT. They recorded the dose length product (mGy*cm) and subsequently used known conversion factors to calculate the effective dose in mSv, which is therefore an estimate [21]. The equivalent dose protocol was run with both systems analyzed, thus avoiding a structural source of error when comparing radiation exposure [26]. Despite the difference in data collection, the results support the findings of the present study.

However, Farah et al. came to a different conclusion in their study comparing iCT and oCBCT, as they measured a lower radiation application of the oCBCT. In contrast, they analyzed the radiation applied by the system by calculating the radiation dose from the dose protocol of the systems, rather than measuring the actual exposure of the patient. Also, on average 3.6 vertebral bodies were instrumented with oCBCT and 4 vertebral bodies with iCT [8]. Therefore, in their study one scan with the oCBCT was sufficient to capture the entire intervention sight. In the present study, 8 instead of 4 vertebral bodies were instrumented per spine model. As mentioned, due to the limited field of view two refencing and two evaluation scans were necessary with the CBCTs, which could explain the different results.

In their study, Tajsic et al. report lower radiation exposure by cCBCT than by oCBCT. Similar systems were studied since the previous model of the cCBCT used in the present study and the same oCBCT were analyzed. However, unlike in the present study, calculated dose values based on device protocols were compared. Nevertheless, the result of Tajsic et al. endorses the results found in this study [22].

Nachabe et al. also investigated the radiation exposure of an oCBCT (O-Arm, Medtronic, Minneapolis, USA) and XpertCT Augumented Reality Surgical Navigation system for the lumbar spine [28]. They showed that both systems emit less radiation than a conventional postoperative CT scan. They recorded a dose of 15 mGy at the center of their phantom, using a standard oCBCT dose protocol (O-arm, Medtronic, Minneapolis, USA), which is consistent with the mean value measured in the present study. The equivalent radiation exposure was also determined in the same unit as in the present study. Nevertheless, direct comparison is limited because of the discrepancy in data collection and the use of artificial bone models.

In their review, Du et al. investigated the radiation application during screw placement using 2D-based navigation, CT-based navigation, and oCBCT-based navigation [20]. They noticed a greater radiation emission for 2D-based navigation than for CT-based navigation [29]. According to their data, oCBCT-based navigation applied less radiation than CT-based navigation; however, CT-based navigation included preoperative CT in this case and is therefore different from the iCT-based navigation applied in the present study.

Although this study reports a significantly higher radiation dose for oCBCT based navigated pedicle screw placement, time effort was less with this system combination compared to the other system combinations.

Operating time should be kept as short as possible for various reasons [30]. The patient benefits from shorter operating time because it reduces the chances of complications caused by medication applied during anesthesia and potential long-term effect from spinal cord compression. By surveying time effort, this aspect is investigated in our study. In addition, regarding economic aspects, an efficient use of the operating rooms is desired and this can most likely be ensured by reducing the operating time. The comparison of time effort in other research is difficult, since the methods for the measurement of time required for pedicle screw placement is inconsistent. Only trends within studies can be compared, but validity is limited due to the wide variation in data collection [6, 31–33]. For example, in this study, time required for the preoperative “set-up” was not considered because artificial partial spine models were studied. Therefore, a direct translation of the absolute time required for surgery into clinical practice cannot succeed. Nonetheless, the comparison of the systems within this study can be considered expressive, as the all testing followed the same standardized procedure.

## Limitation

The measured dose values are not directly transferable to clinical practice, since absorption of any radiation by soft tissues is unaccounted for due to the use of artificial bone models. The differences in the visualization of the spine segment led to an unequal number of scans in the groups depending on the imaging system used. This must be taken into account when interpreting the radiation dose measurements and comparisons. Also, the location of the dosemeters might add a bias towards a imaging system, since the position of the dose meter within the scan may have an effect on radiation dose registered. The exact extent of regulation of dose performed by the imaging systems was not assessed by the authors. The “set-up” time was not included in this study, but should be considered in the design of future studies.

## Conclusion

The results of this study show that more than twice the radiation dose can be measured for the use of oCBCT based navigation compared to cCBCT based navigation, even though the same number of scans is necessary. Yet, time effort was significantly less for oCBCT based navigation. The larger field of view of the iCT might pose an advantage for dorsal instrumentation of more than five vertebrae. These results pose a potential clinical relevance. Experimental cadaveric and clinical studies should be performed comparing the systems in a standardized fashion, since the results of this study cannot be directly transferred to the clinical setting. Also, other clinically relevant aspects including anatomy, pathogenesis, accuracy of screw placement and secondary diseases of the patient; as well as workflow specific parameters like time demands with regard to the preoperative preparation phase, usability and learning curve should be analyzed.

## Data Availability

The datasets generated and/or analysed during the current study are not publicly available due the size of the data but are available from the corresponding author on reasonable request.

## Abbreviations

CT: Computertomography
CBCT: Cone Beam Computertomography
cCBCT: C-arm Cone Beam Computertomography
oCBCT: O-arm Cone Beam Computertomography
iCT: Intraoperative Computertomography
3D imaging: Three dimensional imaging
2D imaging: Two dimensional imaging
T11: Thoracal vertebra 11
L1,4,5: Lumbar vertebra 1,4,5
S1: Sacral vertebra 1
IEC: International Electrotechnical Committee
ANOVA: Analysis of Variance
SD: Standard deviation
